# Improving risk perception and uptake of pre-exposure prophylaxis (PrEP) through interactive feedback-based counselling with and without community engagement in young women in Manicaland, East Zimbabwe: study protocol for a pilot randomized trial

**DOI:** 10.1186/s13063-019-3791-8

**Published:** 2019-12-02

**Authors:** Ranjeeta Thomas, Morten Skovdal, Matteo M. Galizzi, Robin Schaefer, Louisa Moorhouse, Constance Nyamukapa, Rufurwokuda Maswera, Phyllis Mandizvidza, Timothy B. Hallett, Simon Gregson

**Affiliations:** 1 Department of Health Policy, London School of Economics and Political Science, Cowdray House, London, WC2 2AE UK; 2 Section of Health Services Research, Department of Public Health, University of Copenhagen, Øster Farimagsgade 5 opg. B, Postb, 15, Building: 15.0.17, 1014 København K, Denmark; 3 Department of Psychological and Behavioural Science, London School of Economics and Political Science, London, WC2 2AE UK; 4 Department of Infectious Disease Epidemiology, Imperial College London, St Mary’s Campus Norfolk Place, London, W2 1PG UK; 5 Biomedical Research and Training Institute, 10 Seagrave, Avondale, Harare, Zimbabwe

**Keywords:** Pre-exposure prophylaxis, HIV prevention, Randomized trial, Zimbabwe

## Abstract

**Background:**

HIV incidence in adolescent girls and young women remains high in sub-Saharan Africa. Progress towards uptake of HIV prevention methods remains low. Studies of oral pre-exposure prophylaxis (PrEP) have shown that uptake and adherence may be low due to low-risk perception and ambivalence around using antiretrovirals for prevention. No evidence exists on whether an interactive intervention aimed at adjusting risk perception and addressing the uncertainty around PrEP will improve uptake. This pilot research trial aims to provide an initial evaluation of the impact of an interactive digital tablet-based counselling session, correcting risk perception, and addressing ambiguity around availability, usability, and effectiveness of PrEP.

**Methods/Design:**

This is a matched-cluster randomized controlled trial which will compare an interactive tablet-based education intervention against a control with no intervention. The study will be implemented in eight sites. In each site, two matched clusters of villages will be created. One cluster will be randomly allocated to intervention. In two sites, a community engagement intervention will also be implemented to address social obstacles and to increase support from peers, families, and social structures. A total of 1200 HIV-negative young women aged 18–24 years, not on PrEP at baseline, will be eligible. Baseline measures of endpoints will be gathered in surveys. Follow-up assessment at six months will include biomarkers of PrEP uptake and surveys.

**Discussion:**

This will be the first randomized controlled trial to determine whether interactive feedback counselling leads to uptake of HIV prevention methods such as PrEP and reduces risky sexual behavior. If successful, policymakers could consider such an intervention in school-based education campaigns or as post-HIV-testing counselling for young people.

**Trial registration:**

Clinicaltrials.gov, NCT03565575. Registered on 21 June 2018.

## Background

HIV incidence remains extremely high in adolescent girls and young women (AGYW) aged 15–24 years in sub-Saharan Africa, accounting for one in five new HIV infections in 2017 despite representing just 10% of the population [1]. The urgency to reduce HIV incidence among AGYW is widely recognized. The United Nations General Assembly adopted a Political Declaration with the target of < 100,000 new infections in AGYW by 2020 [2]. In Zimbabwe, where 9000 AGYW became newly infected with HIV in 2017, the Zimbabwe National HIV and AIDS Strategic Plan aims to halve HIV incidence by 2020 [3].

A number of efficacious HIV prevention methods exist that could reduce HIV incidence for AGYW either directly (e.g. pre-exposure prophylaxis [PrEP], condoms, partner reduction) and indirectly (e.g. male partner reduction, voluntary medical male circumcision [VMMC]) but use of these strategies is still low [4]. PrEP, in particular, can empower individuals with few other personal prevention options and so has the potential to strongly reduce HIV incidence among AGYW [5, 6]. However, PrEP remains largely unavailable in sub-Saharan Africa and slow progress has been made towards the UNAIDS 2020 target of reaching 3 million people at risk of HIV with PrEP [5].

Oral PrEP has demonstrated substantial HIV prevention benefits (up to 75% reduction in HIV incidence) in trials. Qualitative evidence from the VOICE trial indicates that low uptake and adherence may be due to uncertainty and ambivalence about using antiretrovirals for prevention and concerns about side effects [7]. In addition, the FEM-PrEP study found that women underestimated their risk of infection and that perceived risk was associated with greater adherence to PrEP [8]. A key gap is whether, when counselled about their risk of infection, the local availability, effectiveness, tolerability, and ease of use of PrEP, AGYW in Zimbabwe will take up oral PrEP.

Two previous studies have evaluated the impact of disaggregated information on HIV risks. Datta et al. [9] evaluated the impact of an interactive computer game on changes in respondents’ (men and women aged 15–19 years) understanding of the relationship between HIV-risk and age, and retention of knowledge after three months. Dupas [10] evaluated a classroom intervention (women in grade 8) providing disaggregated risk information to young women and used teen pregnancies as an objective proxy for unprotected sex. These studies, however, did not consider long-term behavior change, for example, partner reduction or uptake of HIV prevention methods. In the case of Datta and Burns [9], the sample consisted of university students so may be of limited generalizability.

The present study makes several innovative contributions. It tests the usability and acceptability of interactive learning delivered through tablets in urban and rural populations in a sub-Saharan African setting. In addition, it is the first study to evaluate longer-term behavior change resulting from interactive feedback-based learning regarding relative HIV risk. It evaluates the impact of such learning on uptake of PrEP, a relatively new HIV prevention method in Africa.

The purpose of this study is to pilot test and evaluate the impact on risk perception and PrEP uptake within six months in AGYW aged 18–24 years of an interactive digital tablet-based counselling session, aiming at correcting risk perception and addressing ambiguity around availability, usability, and effectiveness of PrEP. The study hypothesizes that correcting misperceptions of risks of HIV infection and offsetting ambiguity effects about the availability, usability, and efficacy of PrEP, through localized, interactive, tablet-based counselling, will improve risk perception and increase uptake of PrEP in HIV-negative AGYW without having an adverse effect on underlying sexual risk behavior.

## Methods/Design

### Study design

**Fig. 1:**
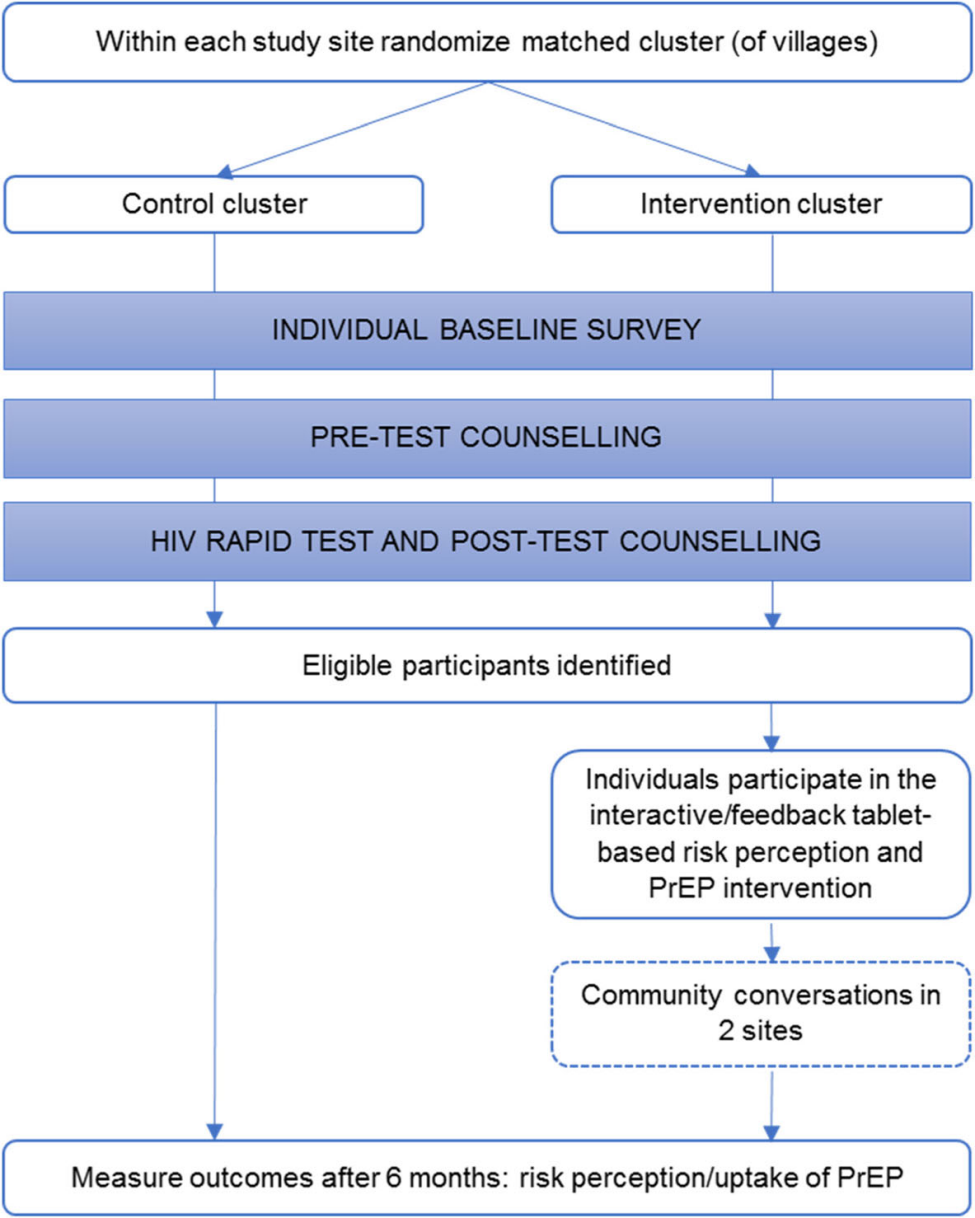
Study design

This is a prospectively registered, two-arm matched-cluster randomized controlled trial (RCT) with single (outcomes assessor) masking, intention-to-treat analysis, and six-month follow-up (Fig. 1). The study is being implemented in eight sites in Manicaland Province, east Zimbabwe. The study will be implemented consecutively on a site-by-site basis. In each site, two matched clusters of villages will be created. Clusters will be randomly allocated to intervention or control arm. In each study site, a census of all households in the study clusters at baseline will be used to identify AGYW aged 15–24 years who are resident within the study areas. A detailed individual questionnaire will collect information on primary endpoints, following which provider-initiated HIV testing and counselling will be offered. Individuals identified as eligible following this stage will be invited to participate in the intervention.

Intervention participants will play an interactive digital tablet-based quiz including the following information components: (1) risks of HIV infection under different scenarios such as between two hypothetical individuals and with different behaviors; and (2) interactive counselling on key facts around PrEP, its usability, and local availability. Participants will have the option to sign up for contact by a nurse from the health clinic to discuss PrEP further and to make an appointment for a visit. In addition, in two of the eight sites, a community-engagement intervention using Community Conversations (CC) [11, 12] will be implemented to address social and cultural barriers to taking up PrEP and support the abovementioned individual-level intervention. No interventions will be implemented in the control arm.

Participant informed consent and data collection will be carried out by trained research assistants. HIV testing will be done by appropriately trained and accredited staff following the standard procedures and testing algorithm used in the Zimbabwe Ministry of Health and Child Care (ZMoHCC) routine provider-initiated testing and counselling (PITC) services. HIV field test results will be returned to participants the same day. Participants who test positive for HIV will receive referral to treatment services in accordance with national guidelines. Participants testing negative for HIV will also receive post-test counselling and, in intervention clusters, will be invited to participate in the intervention.

Intervention participants will be referred to clinics offering PrEP. When individuals begin PrEP in the form of Truvada (a fixed combination of Tenofovir-TFV and Emtricitabine-FTC), adherence to the once-daily intake of the drug will be monitored using the levels of TFV as a biomarker [13]. This will give direct quantitative measures of adherence and will enable appropriate interventions to be implemented to reduce both the risk of sero-conversion and the risk of developing drug resistance to the acquired infection. Dried blood spots (DBS) will be collected from participants six months after initiation of Truvada-based PrEP.

Statistical analyses will be carried out by researchers blinded to group allocation. Before enrolment, all participants will provide written informed consent. All personal data will be confidential. The study has obtained ethical approvals from the Medical Research Council of Zimbabwe (Ref. MRCZ/A/2243), the institutional review board of the Biomedical Research and Training Institute in Zimbabwe (Ref. AP140/2017), and the Imperial College London Research Ethics Committee (Ref. 17IC4160).

### Qualitative explorations

Formative qualitative explorations will be embedded into different stages of the study. Formative consultations with local stakeholders and a youth advisory board will provide feedback on the intervention procedures, while qualitative studies will contextualize study outcomes by exploring local norms, experiences and perspectives concerning PrEP. Participants for the qualitative studies will be recruited from settings randomly chosen because of their rural/urban location and for being subject to both PrEP and community conversation interventions – allowing a contrast of experiences. Forty AGYW from the two settings will be invited to participate in either individual interviews (*n* = 12), focus group discussions (*n* = 18), or participatory photography (*n* = 10) with the aim of revealing how they encounter, respond to, and negotiate PrEP uptake and engagement. The AGYW will be chosen from the baseline survey by purposeful sampling following the criteria that they have to be: aged 18–24 years; HIV-negative; sexually active; considered “at risk” (according to a WHO risk screening tool); agreed to be contactable; and volunteered to participate. In addition, AGYW will be sampled to represent a mix of those who attended and did not attend PrEP sessions, to explore their reasons and motivations to participate (or otherwise) in the intervention. Individual interviews with parents of AGYW (*n* = 8), interviews with HIV prevention service providers (*n* = 12), focus groups with community members (*n* = 6; ~ 36 participants), and qualitative studies with 40 young men will also be conducted to gain insight to the broader social context of PrEP and AGYW friendly HIV-prevention services. The proposed sample sizes are estimates deemed sufficient to reach theoretical saturation [14].

### Study setting

**Fig. 2:**
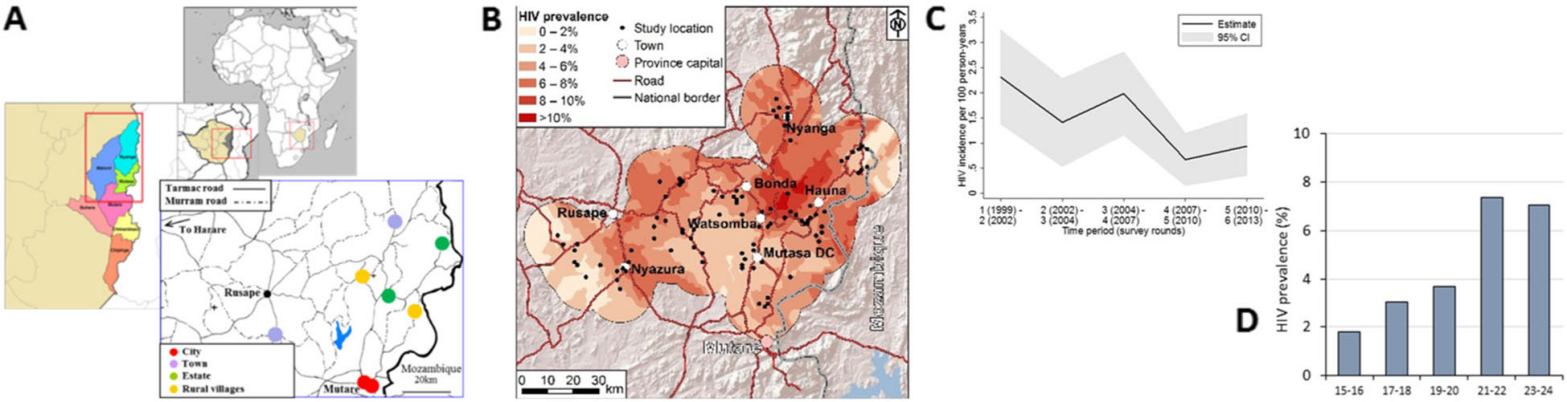
Study sites and patterns of HIV risk in AGYW in Manicaland, Zimbabwe: (**a**) study sites by type and location; (**b**) spatial pattern of HIV prevalence, 2009–2011; (**c**) trends in HIV incidence over time, 1998–2013; and (**d**) HIV prevalence by two-year age group

The study is being conducted in Manicaland province in east Zimbabwe, covering an area of 36,459 km^2^ (14,077 square miles) and has a population of approximately 1.75 million (2012) (Fig. 2). Six sites enumerated in a general population cohort survey that ran from 1998 to 2013 [15]—two small towns, two large agricultural estates, and two rural villages—will be included in the study, together with two new sites in high-density urban suburbs in Mutare, the provincial capital (Fig. 2a). The total estimated population size in these areas is 35,760 people.

Existing data show that, in the rural study sites, HIV prevalence in AGYW (15–24 years) was7.0% (20.6% in women and men aged 15–54 years) in towns and 2.1% (11.9% in women and men aged 15–54 years) in villages in 2012–2013 (Fig. 2b). Figure 2d shows prevalence increases rapidly with age. Prevalence in pregnant women (aged 15–24 years) in Sakubva, a site not previously included in the general population cohort, was 13.4% among AGYW (14.3% in women and men aged 15–54) in 2012 [16].

### Intervention procedures

At the start of the intervention, research assistants will explain the intervention to the treatment arm participants. Participants will be informed that they will be participating in an interactive tablet-based counselling quiz. The sessions will be held in groups of approximately 20 participants.

**Fig. 3:**
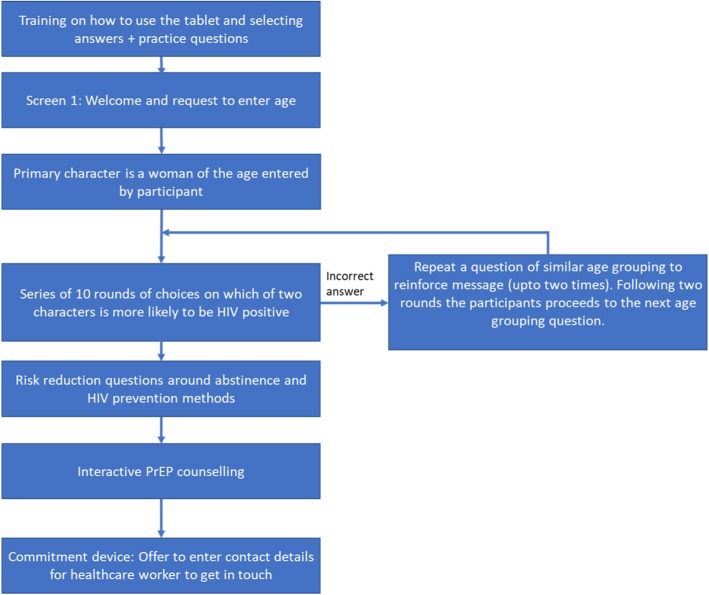
Intervention procedures

The session will begin with training on how to use the tablet and practice questions. Participants will be encouraged to ask questions by raising their hands and the research assistant will answer every question in private. Figure 3 provides an outline of the intervention procedure. Participants will then play the interactive counselling intervention. The information is primarily pictorial and all information is presented in the local language. The interactive counselling will include the following information components:

- Interactive communication of the risks of HIV infection under different scenarios:
  • In each round, participants will be presented with a question asking which one of two hypothetical individuals was more likely to have HIV;
- They will also be asked to choose between different behaviors (e.g. abstinence or condom use) to reduce the risk of acquiring HIV;
- Interactive counselling on key facts around PrEP including: what is PrEP; who is PrEP for; how is PrEP taken; potential side-effects; and availability.

In all cases, participants will immediately be provided with feedback on the accuracy of their choice, the correct answer, and a reinforcing message such as “HIV prevalence is higher amongst older men.”

Following insights from behavioral economics, the study will also implement a non-binding commitment device to enhance participants’ engagement. In particular, all individuals will be encouraged to provide their contact numbers and a preferred time for contact by a nurse from the health clinic to discuss PrEP further and make an appointment for a visit.

**Fig. 4:**
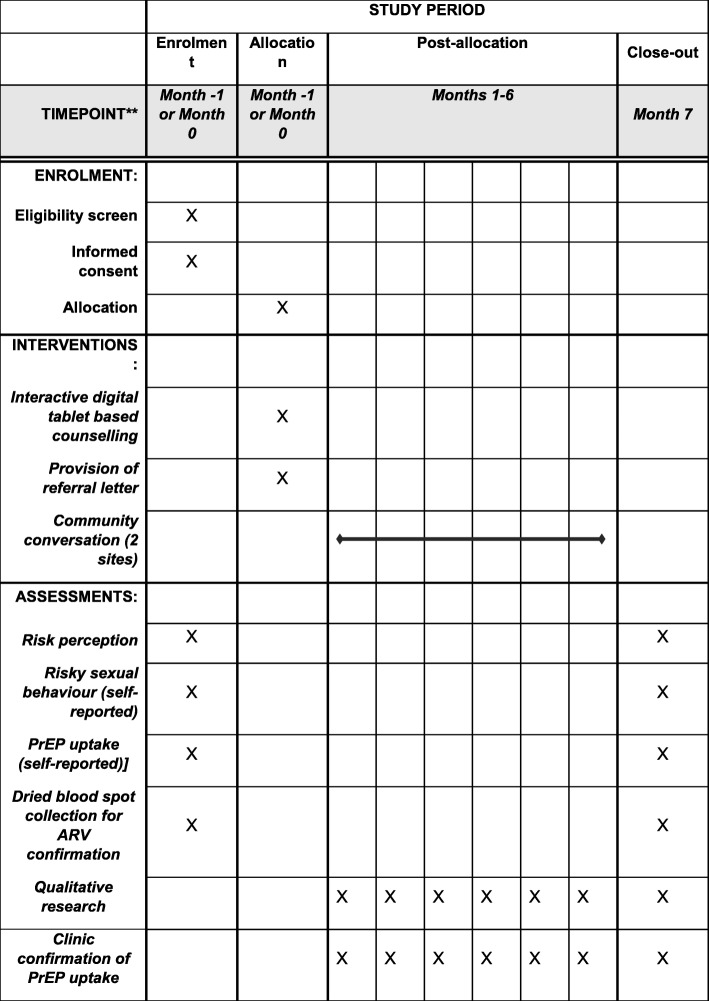
Timeline of study procedures

Participants will also be provided with a PrEP referral letter that they can present directly at one of the participating study clinics to begin PrEP. This article adhered to the Standard Protocol Items: Recommendations for Interventional Trials (SPIRIT) 2013 checklist (see Additional file 1 and  Fig. 4).

#### Further procedures for clusters with community-led interventions

A participatory reflection and action (PRA) approach to community engagement will be used. The approach of involving local community structures in addressing health challenges is based on strong theoretical foundations [17, 18] and has been applied successfully in other health programs [19–21] and to tackle gender-based violence in the context of HIV [22]. This approach supports critical thinking and problem solving around key community issues through CCs. CCs involve posing questions and thinking points to formal community groups or informal networks around community challenges and about potential local responses and community resources to tackle these. In Zimbabwe, CCs have helped to raise community HIV competence [23]. This study will be the first to explore whether and how CCs—as a community engagement strategy—can break down community-level barriers with implications for uptake of PrEP. In these clusters, the Diocese of Mutare Community Care Programme (DOMCCP) will initiate and support a process to: (1) mobilize and work with pre-existing village structures and local community groups who have demonstrated a commitment to fight HIV [24]; (2) conduct CCs with formal groups at village level, generating community action plans; and (3) provide modest funding and technical support to community groups to implement their planned activities. A toolkit on CCs for HIV prevention has been developed to assist the facilitators, standardizing the process and ensuring that CCs stay focused on HIV prevention strategies for AGYW and male partners. The toolkit has been designed to complement the PrEP intervention by: (1) instigating discussions on HIV risk – cementing the salience of HIV prevention methods and on social preferences and biases to use of PrEP; (2) identifying innovative community-led solutions that help AGYW and young men overcome social barriers and re-think risk perceptions; and (3) developing clear steps for communities to prevent the spread of HIV, drawing on local knowledge, resources, and existing local activities. Practically, each CC will comprise 10–15 people who, in four sessions, will: (1) share personal experiences; (2) discuss the challenges/factors preventing AGYW and partners from using HIV prevention methods; (3) explore how to respond; and (4) agree on steps to include in action plans and identify the necessary resources. The community groups will then implement, evaluate, and update their plans.

### Inclusion and exclusion criteria

A total of 1200 women will be considered eligible if: (1) they are aged 18–24 years; (2) they live in the intervention cluster (see below) in one of the eight study sites in Manicaland province; and (3) they expect to be resident there for the duration of the study. Participants will be excluded if they test positive for HIV at baseline or self-report taking PrEP at baseline. To avoid indirect disclosure of the status of HIV-positive individuals, a random sample of 90% of otherwise eligible HIV-negative women will be selected to participate in the intervention.

### Randomization/masking

In each study site, community leaders will first be contacted and clearance will be sought to carry out activities. In addition, community meetings will be organized to introduce the activities before the start of any procedures. Within each of the eight study sites (including the two sites with the additional CC intervention component), detailed site maps will be used to create two matched clusters of villages based on socioeconomic characteristics and factors such as distances to trading centers, major roads, and health centers. Clusters within a site will be randomly allocated to intervention or control using a simple coin toss process. Thus, all eight sites will contain two clusters each (intervention and control). Young women in intervention clusters will receive the interactive intervention (and in two sites, CCs will also be implemented). Young women in the control villages will receive no intervention and will be followed up for measurement of outcomes at six months. Participants will not be masked for the intervention they receive due to cluster level randomization and the nature of the intervention. Study interventions and measurements will occur in separate locations and time points thus facilitating the blinding of outcomes assessors.

### Primary and secondary outcomes

HIV prevention cascades (HPCs) have been proposed to facilitate identification of gaps in implementation of primary HIV prevention methods, understanding of the reasons for these gaps, and identification and evaluation of promising interventions to address the gaps [25, 26]. The population of HPCs using existing study data motivated the selection of some of the outcomes for this study. While no prior measurements were available to populate a HPC specifically for PrEP, analyses of the HPC for existing prevention methods among AGYW in the study population [25, 26] found major gaps in perceptions about personal risk of HIV infection.

The primary outcome for the study will be the proportion of women taking up PrEP within six months measured via biomarkers of ARV presence from DBS and triangulated with self-reports and clinic data. The secondary outcomes will be risk perception measured at baseline and in a follow-up survey at six months, and self-reported change in sexual behavior between baseline and follow-up surveys.

### Sample size estimates

It is estimated that a sample size of 600 participants per arm would be necessary to detect meaningful minimum effect sizes (around 20%) for the primary endpoint (PrEP uptake), with 80% power (*α* = 0.005). Survey data from the study sites in 2012–2013 were used to calculate mean cluster size, coefficient of variation in cluster size (0.15 for women), and the intra-cluster correlation coefficient (ICC) (0.04 using HIV incidence as the outcome). Baseline prevalence estimates of endpoint indicators were assumed to be 10% for PrEP. It was also assumed that 80% of eligible participants consent to participate and that 80% of these are followed up at six months (mean cluster size of 48). The exploratory nature of the intervention combining interactive counselling and community engagement means that we have chosen to use only two clusters for this intervention as this allows greater power to detect the effect without the community-level intervention. Nevertheless, in the two sites with the combined intervention, the study is powered to detect large impacts (effect size difference = 40%) on the outcome indicators. As a further consequence, there is not sufficient power to examine the interaction of the effect sizes with and without the addition of the community-level intervention.

### Statistical analysis

Analysis of the primary outcomes will be done on an intention-to-treat basis. For each endpoint, a linear regression will be used to estimate differences in the cluster-level prevalence of the indicator at follow-up. The intervention and control clusters in the six study sites with only the digital interactive counselling will be compared in one analysis. A separate analysis will compare intervention and control clusters in the two study sites which also include the community conversations component. Any indicators unbalanced at baseline will be included as covariates. The Shapiro–Wilk normality test will be used to test for non-normality of the model residuals, and standard non-parametric tests (e.g. Mann–Whitney rank-sum test) will be used in case of non-normality. Effect sizes will be reported with 95% confidence intervals and results will be considered significant if *P* < 0.05. Data will be analyzed using STATA version 15.

## Discussion

This trial will evaluate the impact of an interactive counselling game on improving perception of HIV risk and uptake of PrEP among young women aged 18–24 years. The present study will provide reliable and high-quality estimates of the average treatment effects because it is a randomized and prospectively registered study, it masks the evaluators, and it uses an intention-to-treat approach. Sample size was calculated to provide adequate statistical power to identify possible differences in the study’s primary outcome. In addition, the six-month follow-up will allow adequate time for participants to present at clinic with referral letters for PrEP and to evaluate changes in risky sexual behavior but not be too long to measure adjustment in risk perception. Findings from this study will help determine whether interactive feedback counselling leads to uptake of HIV prevention methods such as PrEP and reduces risky sexual behavior. If successful, policymakers could consider such an intervention in school-based education campaigns or as post-HIV-testing counselling for young people.

This study has limitations. Some participants may be unable to read information presented as text, although the general literacy level in Zimbabwe is relatively high even in rural areas. Another potential limitation is the possibility of lower than expected retention rates especially among young women who may move away due to marriage or for family reasons. Any retention challenges will provide valuable information in planning for future research and intervention implementation. Other limitations of this RCT could include recruitment challenges and potential sampling bias. The eligibility, recruitment, enrolment, and retention will be closely monitored to overcome such challenges.

To reach and maintain the UNAIDS 90–90-90 goals and to achieve the Sustainable Development Goal of ending the AIDS epidemic as a public health threat by 2030, considerable expansions of HIV prevention efforts are needed. This is especially the case for AGYW who face a high risk of acquiring a lifelong incurable infection which threatens their health and wellbeing. However, while efficacious methods of HIV prevention exist, their uptake remains limited. This study will generate important knowledge to inform HIV prevention policies about the effectiveness of novel interventions to improve HIV prevention uptake, which are devised in such a way that they could be easily scaled up (e.g. as an extension to routine HIV testing and counselling services). These results will not only be applicable to Zimbabwe but also to other settings with high HIV prevalence.

## Trial status

This trial has been registered on clinicaltrials.gov (identifier: NCT03565575) on 21st June 2018. https://clinicaltrials.gov/ct2/show/NCT03565575. Recruitment commenced on 7 July 2018 and is expected to be completed by 31 December 2019. Follow-up data collection will be completed by 31 August 2020. Protocol version 1.6, 19 April 2019.

## Supplementary information

**Additional file 1.** SPIRIT 2013 Checklist: Recommended items to address in a clinical trial protocol and related documents*.

## Data Availability

The datasets generated and/or analyzed during the current study will available from the research team on reasonable request. In addition, following the completion of the study, the authors plan to make data available by request from the Manicaland Centre for Public Health website www.manicalandhivproject.org.

## Abbreviations

AGYW: Adolescent girls and young women
CC: Community Conversations
DOMCCP: Diocese of Mutare Community Care Programme
HIV: Human immunodeficiency virus
HPC: HIV Prevention Cascade
PRA: Participatory reflection and action
PrEP: Pre-exposure prophylaxis
VMMC: Voluntary medical male circumcision
ZMoHCC: Zimbabwe Ministry of Health and Child Care
